# Prevalence and Associated Factor of *Schistosoma Haematobium* Among Primary School Children in Dubti City District, Afar, Northeast Ethiopia, 2022. “A Cross‐Sectional Study”

**DOI:** 10.1002/hsr2.72430

**Published:** 2026-04-26

**Authors:** Setitual Mesfin, Osman Ahmed, Ayenew Berhan, Andargachew Almaw, Dessie Tegegne, Getaneh Alemu, Yehunie Yirdaw, Getu Abeje

**Affiliations:** ^1^ Department of Nursing, College of Medical and Health Science Samara University Samara Ethiopia; ^2^ Department of Medical Laboratory Science, College of Health Science Debre Tabor University Tabor Debere Ethiopia; ^3^ Department of Medical Laboratory Science, College of Medical and Health Science Bahir Dar University Bahir Dar Ethiopia; ^4^ Department of Pediatrician and Child Health, School of Medicine Debre Tabor University Tabor Debere Ethiopia

**Keywords:** Afar, Ethiopia, infection, S. heamatobium, school children

## Abstract

**Background and Aims:**

Human schistosomiasis is a parasitic disease causing organ damage and developmental delays in children. Updated data on its distribution is vital for targeted interventions. This study aimed to determine the prevalence and associated factors of *Schistosoma haematobium* infection among school‐age children in Dubti city district, Northeast Ethiopia.

**Methods:**

A school‐based cross‐sectional study was conducted from December 2021 to January 2022. A total of 416 school children were recruited using systematic random sampling. Data on socio‐demographic and environmental factors were collected via a structured questionnaire. Urine samples were analyzed using sedimentation and filtration techniques. Data were analyzed using IBM SPSS Statistics version 23, employing descriptive statistics and both univariate and multivariate logistic regression to identify associated factors.

**Results:**

Of the 405 samples analyzed, the prevalence of *S. haematobium* was 60.2% (95% CI: 55.41%, 64.90%). The prevalence of *Schistosoma haematobium* using two diagnostic techniques, with the filtration method 244 (60.2%) showing significantly higher sensitivity than the sedimentation method 161 (39.8%). Multivariable analysis revealed that sex (AOR = 0.568), residence (AOR = 0.490), swimming habits (AOR = 2.079), washing clothes near drainage (AOR = 2.68), and the source of water for domestic purposes (AOR = 3.311) were statistically significant factors associated with infection.

**Conclusion:**

The study found a high prevalence of *S. haematobium* among school children. To combat this, interventions should focus on providing safe water, conducting mass drug administration in schools, and implementing health education programs.

**Abbreviations**
ANRS: Afar National Regional State
COVID‐19: Corona Virus Disease of 2019
G: Graph
MDA: Mass Drug Administration
Ml: Milli Litter
RPM: Revolutions Per Minute
Neglected: Tropical Diseases
SNNPR: Southern Nation Nationalities region
SH: *Schistosoma haematobium*
SPSS: Statistical Package for the Social Sciences
WHO: World Health Organization.

## 1 Introduction

Schistosomiasis, commonly referred to as bilharzia or snail fever, is one of the oldest known human infections. It is a chronic and debilitating neglected tropical disease caused by water‐borne digenetic trematodes from the genus *Schistosoma*. The five species of medical significance include *Schistosoma haematobium*, *S. mansoni*, *S. japonicum*, *S. mekongi*, *S. guineensis*, and *S. intercalatum* [1, 2], with the first three being the most pertinent from a global health perspective [3, 4]. Transmission of schistosomiasis has been documented in 91 countries globally [5], making it the second most prevalent parasitic disease in the world, following malaria [6].

Among these, urogenital schistosomiasis, caused by the trematode *Schistosoma haematobium*, remains one of the most prevalent neglected tropical diseases (NTDs) in sub‐Saharan Africa. It accounts for nearly half of the morbidity and about 150,000 deaths annually, contributing to significant health burdens, particularly when compared to *S. mansoni* in Ethiopia [7, 8].

The infection is characterized by chronic inflammation of the urogenital tract, leading to hematuria, dysuria, and in advanced cases, irreversible complications such as hydronephrosis and bladder squamous cell carcinoma [3]. Ethiopia bears a significant burden of both intestinal and urogenital schistosomiasis, with transmission occurring across several regional states where ecological conditions favor the intermediate host snails [5, 6]. A comprehensive systematic review and meta‐analysis estimated the national pooled prevalence of schistosomiasis at 22%, though substantial regional variations exist [7].

The epidemiology of *S. haematobium* is particularly concerning in lowland areas below 800 meters altitude, which characterize much of the Afar region [8]. In the Afar region specifically, emerging evidence indicates a substantial burden, with one multi‐regional study documenting a prevalence of 27.3% among school‐age children [9]. Similarly, in the middle part of the Awash Valley, particularly along the floodplains of the Awash River, infection rates of *S. haematobium* have been reported to be between 6% and 52% among semi‐nomadic Afar communities, and between 0% and 27% among agricultural groups [10].

The infection affects people across all age groups, but primary school‐aged children bear a disproportionately higher burden of the disease, with prevalence typically peaking between the ages of 8 and 15 years [11]. This is largely due to their low level of resistance and high levels of water contact during activities such as playing and swimming, making school‐aged children the most heavily infected demographic [11]. The consequences of infection in this age group extend beyond immediate morbidity, encompassing anemia, malnutrition, growth retardation, and impaired cognitive development, ultimately affecting educational attainment and long‐term productivity [3]. Studies conducted in various Ethiopian settings have reported prevalence rates ranging from 12.2% to 43.8% among children, highlighting the focal and heterogeneous nature of transmission [12, 13]

Multiple factors contribute to *S. haematobium* transmission and infection risk. Behavioral factors, particularly water contact patterns such as swimming, bathing, and washing clothes in open water bodies, have been consistently identified as primary risk determinants [14]. Additionally, demographic characteristics, including sex and residence location, along with environmental factors such as proximity to snail‐infested water sources, significantly influence infection patterns [15].

Despite the ongoing progress of numerous water development projects in various low‐altitude areas of Ethiopia, which create favorable conditions for urogenital schistosomiasis, there has not been a comprehensive study that critically examines the magnitude and associated risks of this disease. Previous research has indicated that the distribution of urogenital schistosomiasis in the country is highly localized, primarily affecting lowland areas such as the north‐eastern region, the middle and lower Awash Valley, as well as the eastern region around the lower Wabe Shebelle valleys and Kurmuk near the Ethio‐Sudan border [16, 17, 18].

However, there is a lack of adequate and up‐to‐date epidemiological studies that detail the current prevalence and contributing factors of *S. haematobium* across Ethiopia, particularly in the Afar Regional State. The absence of this local information leads to a lack of awareness among health professionals and the community regarding the disease, which is a significant factor contributing to its continued undiagnosed presence in many regions of the country. Therefore, this study aimed to determine the prevalence of *S. haematobium* infection and identify its associated factors among primary school children in Dubti City District, Afar, North‐Eastern Ethiopia, in 2022.

The findings of this study will provide essential epidemiological data on *S. haematobium* for policymakers, the Federal Ministry of Health, and the Afar Regional Health Bureau, assisting in the evaluation of existing control measures. Additionally, it will inform the design of effective prevention and control strategies for *S. haematobium* infections. The results will also enhance clinicians’ ability to diagnose and manage cases more effectively, while simultaneously fostering community awareness about the transmission of urogenital schistosomiasis.

## 2 Methods

### 2.1 Study Area, Design and Period

A school‐based cross‐sectional study was conducted among school children in Dubti City Administration, Afar National Regional State, Northeast Ethiopia, February, 2022. Dubti Town is situated near the Awash River in the Afar National Regional State of North‐Eastern Ethiopia. It is one of the woredas in Zone one of the Afar Region and shares borders with several districts: the Somali Region to the south, Mille to the southwest, Chifra to the west, Administrative Zone 4 to the northwest, Kori to the north, Elidar to the northeast, Asayita to the east, and Afambo to the southeast. The town is located approximately 12 km from Samara, the regional capital.

According to the national census conducted by the Central Statistical Agency of Ethiopia, this woreda has a total population of 65,342, comprising 34,893 men and 30,449 women [19]. The average elevation of the woreda is 503 meters above sea level [20]. The Awash River divides the woreda into northern and southern sections and has a tributary known as the Logia River. Adjacent to the Awash River are the Dubti Marshes, which span an area of 34 by 12 kilometers.

The woreda contains four primary schools, including Plantation Primary School and Farman Primary School, both located near an irrigation project in the Awash Valley. The total enrollment across these schools is 1,765 students, with 985 students attending Plantation Primary School and 780 students attending Farman Primary School.

**Figure 1:**
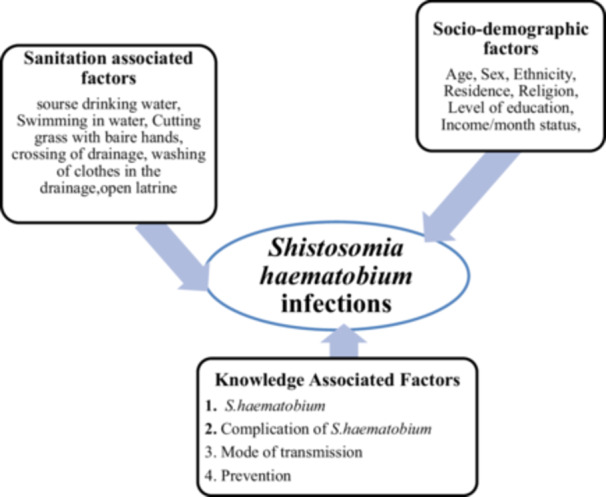
Conceptual Framework Showing the Association between Independent Variables and Outcome Variable (Adapted from Various Literature Sources) [21, 22].

Conceptual Framework showing the association between independent variables and dependent variables (Figure 1).

### 2.2 Eligibility Criteria

Children aged 6 to 14 years who had resided in the woreda town for at least 3 months prior to data collection were eligible to participate. Following the provision of informed consent or assent, they were invited to take part in the study. Children were excluded if they had taken anti‐parasitic medication within the preceding 8 weeks or were absent from school during the data collection period. These criteria were established to ensure the validity and reliability of the study findings.

### 2.3 Sample Size Determination and Sampling Techniques

#### 2.3.1 Sample Size Determination

The sample size for this study was calculated using a single population proportion formula, based on the previous prevalence (p) of *S. haematobium* among school‐aged children residing in the middle and lower Awash Valley, Afar Regional State of Ethiopia, which was reported to be 20.8% [23]. The sample size was determined using the following formula: *N* = (Zα/2) × P(1 − P)/d2. Where: *n* = sample size, *P* = estimated prevalence (20.8%, or 0.208), d = margin of error (0.04) Zα/2 = critical value at 95% confidence level (1.96). Substituting the values into the formula: *n* = 386. After accounting for a non‐response rate of 19 (5%), the final sample size was adjusted to 405. This calculation ensured that the study had a sufficient sample size to achieve reliable results regarding the prevalence of *S. haematobium* among the target population.

### 2.4 Sampling Technique

The target population for this cross‐sectional study encompassed all primary school‐aged children, both enrolled and non‐enrolled, residing in the Dubti City Administration. To establish the sampling frame, a comprehensive list of elementary schools in the Dubti City Administration was obtained from the relevant education office. Two schools were purposively selected for data collection based on their proximity to the Awash River and the presence of irrigation canals and swamps in the area. The study specifically focused on Plantation and Farman Primary Schools due to these specific geographic features.

**Figure 2:**
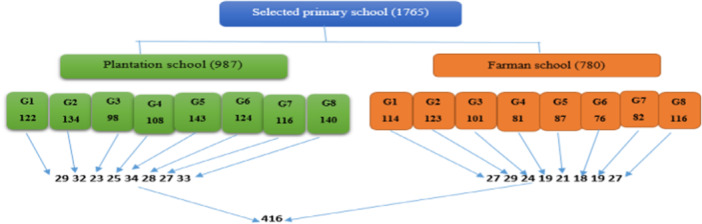
Schematic Presentation of the Sampling Procedure for Selecting Primary School Children in Farman and Plantation Schools, Dubtie District, Northeast Ethiopia, February 2022.

In these selected schools, students from grades 1 through 8 were invited to participate. The sampling process within each class utilized a systematic random sampling technique, with class rosters used as the sampling frame. A starting point was randomly selected using a lottery method, and then every Kth student was included in the study, ensuring a representative sample while reducing selection bias. Ultimately, although 416 children from the two selected schools were initially part of the study, the response rate of 97.4% led to samples being collected from 405 primary school children for the final analysis (Figure 2).

### 2.5 Operational Definition

#### 2.5.1 Definition Statistical Term

In a sample, the *p*‐value, or probability, signifies the likelihood that any observed difference between groups is due to chance. It ranges from 0 to 1. Values close to 1 suggest that there is no significant difference between the groups beyond what might occur by chance, while values near 0 indicate that the observed difference is unlikely to have occurred randomly. Consequently, it is common for medical publications to describe *p*‐values with terms like “very significant” or “highly significant”. The confidence level of 95% means α = 1 − 0.95 = 0.05α = 1 − 0.95 = 0.05. Thus, α/2 = 0.025α/2 = 0.025. We need the *Z*‐score such that the area to its right is 0.0250 (or the cumulative probability from the left is 1 − 0.025 = 0.9751 − 0.025 = 0.975).

K^th^ is a probability sampling method where you select study participants from an ordered list (sampling frame) by choosing a random starting point and picking every K‐th individual (e.g., every 5th or 10th person). The interval ‘k’ is calculated by dividing the total population size (*N*) by the desired sample size (*n*).

**Lugol's iodine solution:** acts as a rapid, non‐specific contrast stain in parasitology to enhance the microscopic visualization of intestinal protozoan cysts, helminth eggs, and larvae in stool wet mounts. It stains glycogen and nuclei brown, aiding in identification, while distinguishing cysts from white blood cells.

Sedimentation (Centrifugation): A common, standard method where urine is centrifuged to force solid materials to the bottom (sediment), allowing the liquid (supernatant) to be removed.

Filtration: Urine is filtered through a membrane of specific pore size to retain all formed elements on the surface, which are then fixed and stained for microscopic examination.

### 2.6 Data Collection

#### 2.6.1 Questionnaire Data

A pre‐tested structured questionnaire in both Amharic and Afarigna was utilized to gather data on the socio‐demographic characteristics of school children and factors perceived to be associated with infection. The questionnaire was administered through face‐to‐face interviews, and the data collection was conducted by trained laboratory technicians. This approach ensured accurate and reliable data regarding the participants’ backgrounds and potential risk factors for infection.

### 2.7 Sample Collection, Processing, and Examination

#### 2.7.1 Urine Sedimentation Technique and Microscopic Examination

Each participating child was provided with guidance on how to collect urine samples and was given two wide‐mouthed, clean urine cups to collect approximately 10 ml of terminal urine in each cup. The urine collected in one cup was processed for examination of urine sediment, while the urine in the other cup was utilized for the filtration technique. The samples were transported to Dubti Referral Hospital in Afar, Ethiopia, within 5 h of collection, using suitable cool boxes maintained at temperatures between 4°C and 6°C for subsequent examination. All urine samples were first tested for hematuria using a dipstick test. Following this, they were examined for the presence of *Schistosoma haematobium* eggs through either ether filtration or a sedimentation method.

To concentrate the eggs of Schistosoma's, 10 ml of terminal urine was centrifuged in a 15 ml conical tube at 2,500 revolutions per minute (rpm) for 5 min. After centrifugation, the supernatant was discarded by gently inverting the test tube, and the sediment was taken. A cover slip was then placed on the concentrated sample, and the preparation was examined under 10× and 40× objectives to detect and identify *S. haematobium* ova. To enhance the sensitivity of urine sediment microscopy, duplicate smears were prepared and examined from each child's sample. This thorough approach ensures a more reliable detection of the infection.

### 2.8 Urine Filtration Technique and Microscopic Examination

A 10 ml sample of vigorously shaken urine was drawn into a syringe and carefully pressed through a 12‐μm polycarbonate filter. The filter was then placed on a microscope slide, and a drop of Lugol's iodine solution was added prior to conducting a quantitative examination under the microscope. It was essential to examine all areas of the filter for accurate quantitative reporting. The eggs found in the sample were counted and recorded as the number of eggs per 10 ml of urine. The intensity of the infection was classified based on the World Health Organization (WHO) guidelines as either light (1–49 eggs/10 ml of urine), moderate (50–499), and heavy (≥ 500 eggs/10 ml of urine) [24, 25]. This classification helps in understanding the severity of the infection within the study population.

### 2.9 Quality Control

A pre‐test was conducted with primary school children at Logia by recruiting 21 students, which represented 5% of the total sample size. This pre‐test aimed to identify any issues related to the questionnaires, sample collection, and processing. Any problems identified during this phase were addressed and corrected prior to the commencement of the actual data collection. Throughout the data collection process, all collected data were checked daily for completeness and consistency. Standard operating procedures were strictly adhered to during the collection and processing of urine samples to ensure reliability.

To ensure the reliability of the study findings, quality control measures were implemented throughout the entire laboratory process, including pre‐analytical, analytical, and post‐analytical phases. Each smeared slide was examined twice by different laboratory personnel for confirmation. Additionally, 10% of the slides from each method were randomly selected and re‐examined at the end of the study by a third laboratory personnel who was blind to the initial examination results. These stringent quality control steps helped to enhance the accuracy and reliability of the study outcomes.

### 2.10 Data Management and Analysis

The data were edited, cleaned, and entered into IBM SPSS Statistics version 23.0.0.0 for analysis. Descriptive statistics, including mean and proportion, were calculated to describe the study participants and estimate the prevalence of *S. haematobium* infection. Univariate logistic regression was employed to evaluate the factors associated with *S. haematobium* infections. Variables with a *p*‐value of less than 0.2 in the univariate analysis were further analyzed using multivariate logistic regression. A factor was considered significantly associated with *S. haematobium* infection if the *p*‐value was less than 0.05, maintaining a 95% confidence level. All tests were two‐tailed.

## 3 Results

### 3.1 Socio‐Demographic Characteristics of Study Participants in Farman and Plantation Primary Schools

A total of 405 primary school children participated in this study, yielding a response rate of 97.4%. The majority of participants were male, comprising 246 individuals (60.7%), while females accounted for 159 individuals (39.3%). Regarding religious affiliation, most participants identified as Muslim (339 participants; 83.7%), with the remaining 66 participants (16.3%) belonging to other religious groups. Notably, the majority of school children (242 individuals; 59.8%) resided in close proximity to the Awash Valley, a known risk factor for schistosomiasis transmission due to potential water contact activities. The socio‐demographic characteristics of the study population are summarized (Table 1).

**Table 1:** Socio‐Demographic Characteristics of Primary School Children in Farman and Plantation Schools, Dubtie District, Northeast Ethiopia, February, 2022.

Variables	Category	*N*	Percent %	Mean (SD)
Sex	Male Female	246 159	60.7 39.3	
Age	5–10 years 11–15 years	157 248	38.8 61.2	11.34 ± 2.654
Education status	1–4 Grade 5–8 Grade	195 210	48.1 51.9	
Religion	Muslim Orthodox	339 66	83.7 16;3	
School type	Plantation Farman	285 120	70.4 29.6	
Income of family	Low (2500 ETB) Average (2501‐8900 ETB)	188 217	46.4 53.67.9	
Residence	Near to Awash Valley Far to Awash Valley	242 163	59.8 40.2	

Abbreviations: ETB, Ethiopian Birr; *N*, number; SD, standard deviation.

### 3.2 Prevalence of *Schistosoma Haematobium* Among Primary School Children Using Filtration and Sedimentation Methods

A total of 405 primary school children were enrolled in this study. The overall prevalence of *Schistosoma haematobium* infection, as determined by the filtration method, was 60.2% (246/405). In this investigation, both the sedimentation and filtration techniques were employed to diagnose *S. haematobium* infections, with the filtration method serving as the reference standard. In contrast, the sedimentation method identified only 161 cases, corresponding to a moderate prevalence of 39.8%. This discrepancy highlights the reduced sensitivity of the sedimentation method observed within the context of this study (Table 2).

**Table 2:** Comparative Prevalence of *Schistosoma haematobium* in Primary School Children Using Filtration Versus Sedimentation Techniques, Dubtie District, Afar Region, Ethiopia, February, 2022.

		Total examined	Number positive	Prevalence (%)
Commulative	Pre *S. haematobium*	405	244	60.2
Methods	Filtration only	405	244	60.2
	Sedimentation only	405	161	39.8

### 3.3 Comparative Infection Intensity of *Schistosoma Haematobium* in Primary School Children Using Filtration Versus Sedimentation Techniques

The diagnostic performance of the two techniques also varied considerably with respect to infection intensity. The mean infection intensity after filtration was 93.9 eggs/10 mL, placing it in the moderate range, though it remained close to the light threshold.

In comparison, the mean intensity after sedimentation was 133.1 eggs/10 mL, also falling within the moderate category. Filtration detected a higher proportion of light infections, 73.8 percent compared to sedimentation's 60.2 percent, which explains why its prevalence estimate was higher overall.

Conversely, sedimentation missed many of those light infections but detected slightly more heavy infections, with 9.6 percent versus filtration's 5.7 percent. These findings underscore the superior sensitivity of the filtration technique, particularly for quantifying high‐burden infections, and highlight the limitations of the sedimentation method for accurate intensity grading (Table 3).

**Table 3:** Comparative Infection Intensity of *Schistosoma haematobium* in Primary School Children Using Filtration Versus Sedimentation Techniques, Dubtie District, Afar Region, Ethiopia, February, 2022.

Method	Examined (*n*)	Positive *n* (%)	Total eggs counted	Mean intensity (eggs/10 mL)	WHO intensity		
					Light (%)	Moderate%	Heavy%
Filtration	405	244 (60.2)	22,900	93.9	180 (73.8%)	50 (20.5%)	14 (5.7%)
Sedimentation	405	166 (41.0)	22,100	133.1	100 (60.2%)	50 (30.1%)	16 (9.6%)

WHO (World Health Organization) intensity class (light: 1–49 eggs/10 mL of urine; Moderate: 50–499; heavy ≥ 500/10 ml of urine.

### 3.4 Prevalence of *Schistosoma Haematobium* Infection Among Primary School Children in Plantation and Farman Primary Schools

The distribution of *Schistosoma haematobium* infection varied between the two participating schools. Of the 244 children diagnosed with schistosomiasis, the majority were from Farman Primary School, accounted for 91 infected children from the total of 120 primary school children, corresponding to 75.8% of the total infections, In contrast, Plantation Primary School accounted for 153 infected children from the total of 285 primary school children, representing 53.7% of all positive cases (Table 4).

**Table 4:** Prevalence of *Schistosoma haematobium* Infection Among Primary School Children in Plantation and Farman Primary Schools, Dubtie District, Afar Region, Northeast Ethiopia, February, 2022.

Name of School	Total examined	Number of positive	Prevalence (%)	AOR	*p*‐value
Plantation	285	153	53.7%	1.866 (0.899, 3.875)	0.065
Farman	120	91	75.8%	2.707 (1.678, 4.368)	0.094

Abbreviation: AOR, adjusted odds ratio.

This disparity suggests differential exposure to cercariae‐infested water sources between the two school populations, potentially attributable to variations in water contact behaviors or proximity to contaminated water bodies such as the Awash Valley.

### 3.5 Water Contact Habit and Toilet Utilization Among Primary School Children In

#### 3.5.1 Plantation and Farman Primary Schools

Among the total study participants, a significant majority reported using water for domestic purposes, particularly through piped water, with 305 individuals (75.3%) indicating this practice. Additionally, the use of toilets in schools was also prevalent, with 308 participants (76%) utilizing these facilities. These findings suggest that access to piped water and school sanitation facilities are common among the study population (Table 5). However, it is important to consider how these factors may influence the overall health and hygiene practices related to schistosomiasis prevention and control within the community. Enhancing the quality of water supply and sanitation facilities can play a crucial role in reducing the risk of infections.

**Table 5:** Water Contact Habits and Toilet Utilization among Primary School Children in Farman and Plantation Schools, Dubtie District, Northeast Ethiopia, February, 2022.

Variable	Category	Number	%
Swimming habits	No	109	26.9
	Yes	296	73.1
Cutting grass in the drainage	No	210	51.9
	Yes	195	48.1
Drainage contact while crossing	No	162	40.0
	Yes	243	60.0
Washing clothes in the drainage	No	178	44.0
	Yes	227	56.0
Water for domestic purpose	Pipe source	305	75.3
	Drainage source	100	24.7
Use toilet in the school	No	97	24.0
	Yes	308	76.0

### 3.6 Knowledge of Schistosomiasis: Symptoms, Transmission, and Prevention

The primary source of information about schistosomiasis for the majority of participants was healthcare facilities, such as clinics or hospitals 69 (37.5%). Regarding clinical presentation, blood in urine (hematuria) was the most frequently reported symptom, identified by 100 participants (82.1%). Furthermore, swimming in infected water was cited as the predominant perceived mode of transmission, with 90 individuals (73.2%).

In terms of prevention, avoiding swimming in drainage areas was the most commonly reported practice among participants, 84 (68.9%). Despite these specific areas of awareness, the overall level of comprehensive knowledge about the disease was concerningly low. A substantial majority of the study population, 360 participants (88.9%), demonstrated poor knowledge regarding schistosomiasis.

These findings underscore a critical gap in understanding that could hinder effective disease control. They highlight an urgent need for enhanced educational programs and community awareness campaigns. Such initiatives should focus on improving knowledge about schistosomiasis, including its symptoms, transmission routes, and preventive measures, to empower the community and effectively combat the infection (Table 6).

**Table 6:** Knowledge, Information Sources, and Awareness of Schistosomiasis (Signs/Symptoms, Complications, Transmission, and Prevention) Among Primary School Children in Farman and Plantation Schools, Dubtie District, Northeast Ethiopia, February 2022.

Variables		Responses		Percent of cases
		*N*	Percent	
Source of information	Health center	20	10.9%	16.3%
	Clinic/hospital	69	37.5%	56.1%
	Media	30	16.3%	24.4%
	School	65	35.3%	52.8%
Sign and symptom	Hesitancy during voiding of urine	73	28.6%	59.3%
	Blood in urine	101	39.6%	82.1%
	Burning sensation	61	23.9%	49.6%
	I do not know	20	7.8%	16.3%
Complication of *SH	Dysuria	53	25.1%	43.8%
	Bloody urine	64	30.3%	52.9%
	Infertility	11	5.2%	9.1%
	Swelling of bladder	22	10.4%	18.2%
	Splenomegaly	11	5.2%	9.1%
	Do not know	50	23.7%	41.3%
Mode of transmission	By open field urination	50	14.7%	40.7%
	By swimming in infected water	90	26.4%	73.2%
	By contact the drainage	61	17.9%	49.6%
	By cutting grass in the drainage	54	15.8%	43.9%
	By washing of clothe in the drainage	53	15.5%	43.1%
	I do not know	33	9.7%	26.8%
Prevention methods	By use of toilet	56	16.0%	45.9%
	Avoiding swimming in drainage	84	23.9%	68.9%
	Avoiding contact with drainage	59	16.8%	48.4%
	Anti‐ schistosomiasis drug	68	19.4%	55.7%
	By cleaning our environment	50	14.2%	41.0%
	I do not know	34	9.7%	27.9%
Knowledge status	Poor Knowledge	360	88.9%	
	Moderate knowledge	35	8.6%	
	Good knowledge	10	2.5%	

Abbreviations: *N*, Number; SH, S*. haematobium*.

### 3.7 *Schistosoma haematobium* Prevalence by Water Contact and Toilet Access

The prevalence of *Schistosoma haematobium* was studied in relation to water contact and sanitation practices among 405 participants. A significant proportion of infected individuals, 210 (86.1%), reported a history of swimming, while 196 (80.3%) consistently used toilets. These results emphasize the impact of these factors on the transmission dynamics of the parasite (Table 7).

**Table 7:** Association Between *Schistosoma Haematobium* Prevalence and Water Contact Habits and Toilet Utilization Among Primary School Children in Farman and Plantation Schools, Dubtie District, Northeast Ethiopia, February, 2022.

Variables		Number of students infected with *Schistosoma heamatobium*	
		*N*	Percent (%)
Swimming habits	Yes (296)	210	86.1%
	No (109)	34	13.9%
Cutting grass near to the drainage	Yes (195)	146	59.8%
	No (210)	98	40.2%
Contact the drainage while crossing	Yes (243)	175	71.3%
	No (162)	69	28.3%
Washing clothes in the drainage	Yes (227)	169	69.3%
	No (178)	75	30.7%
Source of water for domestic purposes	Pipe source (305)	158	64.8%
	Drainage source (100)	86	35.2%
Use of the toilet in the school	Yes (308)	196	80.3%
	No (97)	48	19.7%

Abbreviation: *N*, number.

### 3.8 Factors Associated With *S. Haematobium* Infection

Bivariate logistic regression analysis was performed to investigate the association between various socio‐demographic and behavioral factors and *Schistosoma haematobium* infection among primary school children in Dubtie District. The socio‐demographic factors assessed included age category, sex, religion, residence, school attended, and educational level. Behavioral factors examined included swimming habits, cutting grass near drainage areas, contact with drainage while crossing, washing clothes in drainage water, the source of water for domestic purposes, and toilet utilization at school. These factors were significantly associated with *Schistosoma haematobium* infection.

Variables that demonstrated statistical significance in the bivariate analysis were subsequently included in the multivariable logistic regression model to control for potential confounders. After adjusting for these confounding variables, the analysis identified five factors that remained independently associated with *S. haematobium* infection were: sex, residence near the Awash River, swimming habits, washing clothes in drainage water, and using drainage as a water source.

Female participants exhibited significantly lower odds of *S. haematobium* infection compared to their male counterparts, with being female associated with a 43.2% reduction in the odds of infection (AOR = 0.568; 95% CI: 0.347–0.928; *p* = 0.024). Children living near the Awash River had significantly higher odds of infection compared to those residing further away (AOR = 2.079; 95% CI: 1.175–3.676; *p* = 0.012), highlighting the critical role of proximity to contaminated water bodies as a key risk factor.

Participants who reported swimming in open water bodies had more than twice the odds of infection compared to those who did not swim (AOR = 2.079; 95% CI: 1.175–3.676; *p* = 0.012). Additionally, children who engaged in washing clothes in drainage water faced a significantly increased risk of infection, with odds nearly three times higher (AOR = 2.623; 95% CI: 1.618–4.252; *p* < 0.001). The strongest association was observed among children using drainage water for domestic purposes, who had more than three times higher odds of *S. haematobium* infection compared to those using alternative water sources (AOR = 3.311; 95% CI: 1.703–6.436; *p* < 0.001).

These findings emphasize the need to address environmental and behavioral factors in the prevention and control of *S. haematobium* infections. Public health interventions should focus on educating communities about the risks associated with water contact activities and promoting the use of safe water sources. Additionally, strategies aimed at improving sanitation and reducing exposure to contaminated water will be crucial in mitigating the infection burden in affected populations (Table 8).

**Table 8:** Bivariable and Multivariable Logistic Regression Analysis of Factors Associated With *Schistosoma haematobium* Infection Among Primary School Children in Farman and Plantation Schools, Dubtie District, Northeast Ethiopia, February, 2022.

Variable	Categories	S. *haematobium* status		(COR)	(AOR)	*P*‐value
		Pos (%)	Neg (%)			
Age category	5–10	85 (54.1)	72 (45.9)	1.00	1.00	
	11–15	159 (64.1)	89 (35.9)	1.513 (1.007, 2.274)*	1.168 (0.594, 2.295)	0.653
Sex	Male	165 (67.1)	81 (32.9)	1.00	1.00	
	Female	79 (49.7)	80 (50.3)	0.485 (0.322, 0.730)*	0.568 (0.347, 0.928)**	0.024
Religion	Muslim	214 (63.1)	125 (36.9)	1.00	1.00	
	Orthodox	30 (45.5)	36 (54.5)	0.487 (0.286, 0.829)*	0.620 (0.369, 1.041)	0.071
Residence	Near to Awash valley	179 (74)	63 (26)	1.00	1.00	0.005
	Far to Awash valley	65 (39.9)	98 (60.1)	0.233 (0.153, 0.357)*	0.490 (0.289 0.807)**	
School type	Plantation	153 (53.7)	132 (46.3)	1.00	1.00	
	Farman	91 (75.8)	29 (24.2)	2.707 (1.678, 4.368)*	1.866 (0.899, 3.875)	0.094
Educational Level	G1‐4	109 (55.9)	86 (44.1)	1.00	1.00	
	G 5‐8	135 (64.3)	75 (35.7)	1.420 (0.952, 2.118)*	1.100 (0.570, 2.121)	0.777
Swimming Habits	Yes	210 (70.9)	86 (29.1)	5.386 (3.344, 8.676)*	2.079 (1.175, 3.676) **	0.012
	No	34 (31.2)	75 (68.8)	1.00	1.00	
Cutting grass	Yes	146 (74.6)	49 (25.1)	3.405 (2.233, 5.194)*	1.489 (0.776, 2.859)	0.231
	No	98 (46.7)	112 (53.3)	1.00	1.00	
Drainage contact habit	Yes	175 (72)	68 (28)	3.469 (2.282, 5.273)*	0.849 (0.403, 1.787)	0.667
	No	69 (42.6)	93 (57.4)	1.00	1.00	
Washing clothes in the drainage	Yes	169 (74.4)	58 (25.6)	4.002 (2.626, 6.098)*	2.623 (1.618, 4.252) **	0.000
	No	75 (42.1)	103 (57.9)	1.00	1.00	
Water source for domestic purpose	Pipe source	158 (51.8)	147 (48.2)	1.00	1.00	
	Drainage source	86 (86)	14 (14)	5.717 (3.112, 10.496)*	3.311 (1.703, 6.436) **	0.000
Use the toilet in school	Yes	196 (63.6)	112 (36.4)	1.786 (1.127, 2.832)	1.166 (0.669, 2.034)	0.588
	No	48 (49.5)	49 (50.5)	1.00	1.00	
Ever heard about Schist some	Yes	81 (65.3)	43 (34.7)	1.364 (0.879, 2.116)*	0.976 (0.533, 1.786)	0.936
	No	163 (58)	118 (42)	1.00	1.00	
Knowledge status	Poor knowledge	212 (58.9)	148 (41.1)	6.283 (0.788, 50.12)*	5.341 (0.581, 49.19)	0.139
	Moderate	23 (65.7)	12 (34.3)	1.338 (0.646, 2.773)*	1.119 (0.432, 2.899)	0.817
	Good knowledge	9 (90)	1 (10)	1.00	1.00	

*Note:* Single asterisk (*) indicates statistical significance in bivariable analysis (*p* < 0.05); double asterisk (**) indicates statistical significance retained in multivariable logistic regression analysis after adjusting for confounders (*p* < 0.05).

Abbreviations: AOR, adjusted odds ratio; CI, confidence interval; COR, crude odds ratio; G, Grade.

## 4 Discussions

Schistosomiasis is recognized as one of the most prevalent neglected tropical diseases and continues to pose a significant public health challenge in many developing countries, especially among school‐aged children in sub‐Saharan Africa, including Ethiopia. It severely affects their nutritional status, academic performance, and overall growth [18]. This study aimed to assess the prevalence of *Schistosoma haematobium* and the associated factors among students in primary schools surrounding the “Awash Irrigation Project.” The overall prevalence of *S. haematobium* was determined to be 60.2% (95% CI: 55.41%, 64.90%). This finding indicates that urogenital schistosomiasis constitutes a severe public health problem in the study area, substantially exceeding the threshold for regular mass drug administration interventions as defined by the World Health Organization [26].

This result aligns with studies conducted in the Gambella Region 61.2% among school‐aged children [27]. But the observed prevalence in this study considerably higher than the national pooled estimate of 22% reported in a recent meta‐analysis [28] 55.4% in Southern Nations, Nationalities, and Peoples’ Region (SNNPR) [29], 58.5% in Uganda [30], 56.1% in Nigeria [31], 27.3% reported in Afar and Gambella regions [32], 12.2%, 20.8% in Afar region [12] and 16.7% Gambella region [33].

Conversely, this finding also indicates a lower prevalence rate of *S. haematobium* compared to other studies conducted in different regions of Ethiopia, which reported prevalence rates of 85% in Zarim, 67% in Gorgora, and 67.6% in Finchaa Valley among school children [34, 35, 36]. This discrepancy may be attributed to several factors, including differences in study timing, seasonal variations in transmission intensity, or truly higher endemicity within the Dubti district specifically.

This high prevalence rate highlights a significant public health issue in the region, and the Awash River may also provide a habitat conducive to the life cycle of *S. haematobium*. In addition, frequent exposure to water bodies for activities like bathing, washing, and agricultural practices increases the risk of transmission, as well as its arid climate and scarcity of clean water sources, leading communities to rely on contaminated water for domestic use.

Furthermore, the presence of irrigation systems and natural water bodies can create ideal conditions for the proliferation of *S. haematobium* and its intermediate hosts, primarily freshwater snails as well as traditional practices involving the use of natural water bodies for washing, bathing, and other activities can increase the risk of exposure to infected water, further contributing to the high prevalence of schistosomiasis in the region [37].

The higher prevalence of *S. haematobium* may be due to the proximity of schools to ponds and streams, where children frequently engage in activities such as playing, bathing, swimming, washing, and fetching water for domestic use.

The presence of water bodies where individuals engage in activities such as bathing, washing, and fishing contributes to sustained transmission of the disease. Despite the implementation of mass drug administration (MDA) of praziquantel, high prevalence rates persist among both children and adults in the area. This could be attributed to several factors, including frequent contact with infested water sources means that even treated individuals may be re‐exposed to the cercariae, leading to reinfection. The lower in the prevalence of urogenital schistosomiasis in the study area can be attributed to several key factors.

One significant reason is the drying out of many swamps, which are essential habitats for the intermediate snail hosts of *S. haematobium*, specifically *Bulinus abyssinicus*. As these water bodies diminish, the population of the snail hosts is likely reduced, leading to lower transmission rates of the parasite. Additionally, the distribution of praziquantel, an effective treatment for schistosomiasis, at health posts in the region has likely played a crucial role in decreasing infection rates. By providing accessible treatment options, more individuals can receive necessary medications, further contributing to the reduction of prevalence.

Addressing this public health issue requires targeted interventions, including improving water management, enhancing sanitation infrastructure, and increasing community awareness regarding schistosomiasis prevention. These findings indicate that the challenges faced in Duptie Town are not unique to Ethiopia but are part of a broader public health issue across various African countries.

The prevalence of *Schistosoma haematobium* using two diagnostic techniques, with the filtration method showing significantly higher sensitivity than the sedimentation method. This observed difference is consistent with a body of published research evaluating these methods. The filtration technique's superior detection rate can be attributed to its ability to process a larger volume of urine (typically 10 mL) and concentrate eggs onto a membrane filter for clear visualization. This technique is widely regarded as the gold standard for detecting *S. haematobium* eggs due to its superior sensitivity. By trapping eggs directly on a polycarbonate filter, it facilitates precise quantification, offering a significant diagnostic advantage over less sensitive methods [38]. In contrast, the sedimentation method, while simpler, may result in the loss of eggs during the decanting process, leading to an underestimation of true prevalence. The lower prevalence of 44.4% reported for the sedimentation method in the paragraph mirrors findings from other studies where sedimentation detected fewer cases (e.g., 64 cases) compared to filtration (e.g., 77 cases) [38].

Furthermore, a study in Sudan reported that while the filtration technique had superior sensitivity (43.8%) for detecting *S. haematobium*, it could sometimes produce false‐positive results if filters were not properly washed, a limitation not mentioned in the paragraph [39]. Another critical perspective comes from research advocating for molecular techniques. For example, studies have shown that PCR‐based methods for detecting parasite‐specific DNA in urine sediment are not only more sensitive (99–100%) than conventional methods but can also differentiate between co‐infecting species like *S. mansoni* and *S. haematobium* from a single sample [40, 41]. This suggests that even the 60.2% prevalence found by the filtration method might be an underestimate compared to what molecular methods could reveal.

The multivariate analysis confirmed that specific behavioral and demographic factors were independent predictors of *S. haematobium* infection. The study reaffirms the well‐established association between schistosomiasis and water contact. Swimming in open water bodies was strongly associated with increased infection risk, with children who reported swimming habits having more than twice the odds of infection compared to those who did not (AOR = 2.079, 95% CI: 1.175, 3.676) more likely to be infected, a finding consistent with research from Kenya, where frequent swimming in freshwater bodies was a critical risk factor [42] and this finding is biologically plausible and consistent with extensive literature documenting recreational water contact as a primary transmission pathway for schistosomiasis [31]. Swimming exposes large body surface areas to cercariae‐infested water for prolonged durations, maximizing cercarial penetration opportunities.

Similar associations have been reported in the Gambella region, where children who played or bathed in infested water had nearly three‐fold higher infection odds [43].

Similarly, children who washed clothes near drainage systems having 2.68 times higher odds of infection (AOR = 2.68, 95% CI: 95% CI: 1.618, 4.252), which aligns with previous studies [30]. In addition, households using drainage water for domestic purposes were 3.331 times higher risk of infection than of non‐using drainage water for domestic purposes (AOD = 3.331, 95% CI: 1.703, 6.436).

This finding highlights the importance of domestic water contact patterns in schistosomiasis transmission, particularly among females who typically perform this activity [12]. Drainage systems in urban and peri‐urban areas can create permanent or semi‐permanent water bodies that support snail colonization, especially when they receive slow‐moving, organically enriched water. Our finding corroborates research from other African settings where domestic activities near man‐made water bodies have been identified as significant risk factors [44].

This finding is consistent with research conducted in Nigeria, where the reliance on untreated surface water was strongly linked to increased schistosomiasis prevalence [34].

The finding that children living further from the Awash Valley are 0.490 times less likely to be infected by *S. haematobium* infection, which is supported by similar findings in Ethiopia [27].

Their research highlighted that proximity to freshwater bodies significantly increases the risk of schistosomiasis infection, emphasizing the importance of geographical factors in transmission dynamics. This highlights the need for targeted interventions in high‐risk areas situated near water sources. This finding aligns with research conducted in the Afar Amibera district [45]. Drainage areas within the valley harbor the intermediate snail hosts, significantly increasing the likelihood of infection among school‐age children who frequent these sites. Consequently, children living in close proximity face a heightened risk of both initial infection and reinfection. This risk is particularly pronounced for children near the Awash Valley, who often spend considerable time in these drainage areas assisting with herding or engaging in other outdoor activities outside of school hours, compounding their exposure.

According to the study findings, participants who reported not using school toilets, particularly latrines, exhibited a higher infection burden compared to users. This counterintuitive observation is consistent with research conducted in South Africa and suggests that the use of latrines itself is not directly linked to the mode of transmission. Rather, it may reflect other unmeasured behavioral or environmental risk factors among this group [46].

The likely reason for this pattern is that children residing closer to the Awash drainage spend more time in or near these water bodies, participating in activities like swimming even after school hours. This extended exposure increases their contact with cercariae‐infested water, contributing to higher infection rates.

The analysis showed that male children had significantly lower odds of infection compared to females (AOR = 0.568, 95% CI: [0.34–0.928]. This finding contrasts with several studies that have reported either no gender difference or a higher risk among males [47]. However, our result aligns with observations from some Ethiopian settings where girls may experience greater exposure through gender‐specific domestic responsibilities such as washing clothes and utensils in contaminated water bodies [15]. In many rural Ethiopian communities, girls bear a disproportionate burden of water‐related household chores, potentially increasing their contact frequency with infectious water sources.

## 5 Strengths and Limitations

A major strength of this study is its adequate sample size (405 participants) and the use of the standard urine filtration microscopy technique, which remains the recommended diagnostic method for *S. haematobium* in field surveys [48]. The study provides locally relevant data that can directly inform district‐level NTD control programming. However, several limitations warrant consideration. First, the cross‐sectional design precludes establishing causal relationships between identified factors and infection status. Second, the assessment of behavioral risk factors relied on self‐reporting, which may be subject to recall and social desirability biases. Third, the absence of snail intermediate host surveys limits our understanding of transmission sites and ecological determinants.

Fourth, a single urine examination may have underestimated the true prevalence due to day‐to‐day variation in egg excretion, particularly in light‐intensity infections. Fifth, logistical constraints related to the COVID‐19 pandemic contributed to a non‐response rate and restricted the data collection period, which may affect the robustness of the findings. Sixth, due to resource and time limitations, molecular diagnostic techniques could not be employed, which might have provided a more precise characterization of infection intensity and parasite strains. Finally, the study's restriction to school‐enrolled children may not capture the situation among out‐of‐school children who might face different exposure patterns.

## 6 Conclusion and Recommendations

This study demonstrates an alarmingly high prevalence (60.2%) of *S. haematobium* infection among primary school children in Dubti City District, Afar Region, confirming that urogenital schistosomiasis represents a major public health problem in this community. The identified risk factors, such as swimming habits, washing clothes near drainage, unsafe domestic water sources, and sex, are predominantly modifiable and provide clear entry points for intervention.

Based on these findings, we recommend the following actions:

➣ **Strengthen Mass Drug Administration:** The prevalence substantially exceeds the WHO threshold for high‐risk communities, necessitating annual praziquantel administration to all school‐age children with high coverage.
➣ **Implement Targeted Health Education:** Contextually appropriate health education programs should specifically address the risks associated with swimming in open water bodies and washing clothes near drainage systems, while promoting behavioral alternatives where feasible.
➣ **Improve Water Infrastructure:** The strong association with unsafe domestic water sources (AOR = 3.311) highlights the urgent need for investment in safe water supply infrastructure in Dubti City District, aligning with the water, sanitation, and hygiene (WASH) component of integrated NTD control.
➣ **Conduct Snail Surveys:** Further research should map the distribution of *Bulinus* snails in local water bodies, drainage systems, and potential transmission sites to guide targeted mollusciciding where appropriate.
➣ **Monitor Intervention Impact:** Regular monitoring and evaluation should be established to assess the effectiveness of control interventions over time and detect potential emergence of drug resistance.
  - Finally, the findings of this study underscore the urgent need for intensified, multi‐sectoral interventions combining chemotherapy, health education, and improved water supply to reduce the unacceptably high burden of urogenital schistosomiasis among children in Dubti City District
  - To address this burden, it is essential to ensure a safe water supply in schools and throughout communities to improve the health outcomes of students.
  - Routine urine screening should be implemented as a standard operating procedure in all health facilities across the Afar Region whenever clients visit clinics for other diagnostic purposes.
  - Mass drug administration with praziquantel should be provided to infected patients, alongside efforts to discourage water contact and the use of drainage water for sanitation, particularly in schools, by the Afar Regional Health Bureau.
  - In addition, methods for reducing snail populations, including the application of biocides and other chemicals to prevent snail breeding in the area, should be implemented.
  - Addressing this public health issue requires comprehensive interventions that encompass improved water management, enhanced sanitation infrastructure, and increased community awareness and education regarding schistosomiasis prevention.
  - Further research should focus on the molecular epidemiology and snail distribution of *S. haematobium* around the Awash River in Dubti Town.

## Author Contributions

**Setitual Mesfin:** investigation, writing – original draft, conceptualization, methodology, validation, visualization, project administration, formal analysis, software, data curation, supervision, resources. **Osman Ahmed:** investigation, validation, methodology, software, formal analysis, data curation, supervision, resources. **Ayenew Berhan:** visualization, validation, methodology, software, formal analysis, supervision, investigation, resources. **Andargachew Almaw:** investigation, methodology, validation, visualization, writing – review and editing, project administration, formal analysis, software, supervision. **Dessie Tegegne:** investigation, methodology, validation, formal analysis, visualization, software, data curation, supervision. **Getaneh Alemu:** investigation, visualization, writing – review and editing, validation, formal analysis, project administration, supervision, data curation. **Yehunie Yirdaw:** investigation, methodology, validation, visualization, software, formal analysis, data curation, supervision, resources. **Getu Abeje:** conceptualization, investigation, methodology, validation, visualization, writing – review and editing, software, formal analysis, project administration, data curation, supervision, resources.

## Funding

The authors have nothing to report.

## Ethics Statement

Before commencing the study, ethical approval (project code: 210/2022) was obtained from the Samara University, College of Medicine and Health Sciences Ethical Review Committee. Authorization letters were acquired from APHI, and additional support letters were obtained from Duptie Primary School, the educational bureau, the Duptie district health office, and the Afar Regional Health Office. Study participants and their parents/guardians were briefed on the study's objectives, and written informed consent was obtained from the child's parents or legal guardians. Detailed information about the research purpose, procedures, potential risks and benefits, and data confidentiality was provided to all participants.

Children diagnosed with soil‐transmitted helminth infections received treatment following national protocols and the ethical standards outlined in the Declaration of Helsinki. For participants who were minors or unable to provide consent due to cognitive impairment, consent was obtained from a legally authorized representative or parent/guardian, as applicable. To ensure participant privacy, all personal identifiers were removed from the dataset before analysis. Each participant was assigned a unique study code, and all data were securely stored with password protection.

## Conflicts of Interest

The authors declare no conflicts of interest.

## Transparency Statement

The lead author Getu Abeje affirms that this manuscript is an honest, accurate, and transparent account of the study being reported; that no important aspects of the study have been omitted; and that any discrepancies from the study as planned (and, if relevant, registered) have been explained.

## Data Availability

The original data for this study is accessible through the corresponding author. All authors have reviewed and endorsed the final version of the manuscript, maintaining full access to all study data. They bear complete responsibility for upholding the data's integrity and ensuring the accuracy of the data analysis.

## References

[1] E. M. Abe , W. Guan , Y. H. Guo , et al., “Differentiating Snail Intermediate Hosts of Schistosoma Spp. Using Molecular Approaches: Fundamental to Successful Integrated Control Mechanism in Africa,” Infectious Diseases of Poverty 7, no. 02 (2018 Apr 1): 6–18, 10.1186/s40249-018-0401-z. 29615124 PMC5883423

[2] Y. Hirose , J. Matsumoto , M. Kirinoki , et al., “*Schistosoma Mekongi* and *Schistosoma Japonicum*: Differences in the Distribution of Eggs in the Viscera of Mice,” Parasitology International 56, no. 3 (2007): 239–241, 10.1016/j.parint.2007.03.004. 17521955

[3] C. S. Nation , A. A. Da'dara , J. K. Marchant , and P. J. Skelly , “Schistosome Migration in the Definitive Host,” PLoS Neglected Tropical Diseases 14, no. 4 (2020): e0007951, 10.1371/journal.pntd.0007951. 32240157 PMC7117656

[4] H. G. Bishop , H. I. Inabo , E. E. Ella , and M. Bello , “Urinary Schistosomiasis: Risk Factors and Symptoms Among School Adolescents in Kaduna State, Nigeria,” EUREKA: Life Sciences, no. 2 (2023): 56–62, 10.1056/NEJMra012396.

[5] Mondiale de la Santé, O. and World Health Organization, 2022 , “Schistosomiasis and Soiltransmitted Helminthiases: Progress Report, 2021–Schistosomiaseet Géohelminthiases: Rapport de Situation.” Weekly Epidemiological Record = Relevé Épidémiologique Hebdomadaire (2021). 97, 621–631. 48.

[6] I. Mohamed , S. Kinung'hi , P. N. M. Mwinzi , et al., “Diet and Hygiene Practices Influence Morbidity in Schoolchildren Living in Schistosomiasis Endemic Areas Along Lake Victoria in Kenya and Tanzania—A Cross‐Sectional Study,” PLoS Neglected Tropical Diseases 12, no. 3 (2018): e0006373. 29590175 10.1371/journal.pntd.0006373 PMC5891076

[7] D. P. McManus , D. W. Dunne , M. Sacko , J. Utzinger , B. J. Vennervald , and X. N. Zhou “Schistosomiasis,” Nature Reviews Disease Primers, 4 (1) (2018): 13. View in Scopus. 10.1038/s41572-018-0013-8. 30093684

[8] M. B. Abdel‐Naser , A. Altenburg , C. C. Zouboulis , and U. Wollina , “Schistosomiasis (Bilharziasis) and Male Infertility,” Andrologia 51, no. 1 (2019): e13165, 10.1111/and.13165. 30276841

[9] E. J. Fok , M. Kedir , D. Getachew , and G. Tasew , “Prevalence of *Schistosoma Haematobium* Infection Among School‐Age Children in Afar and Gambella Regions, Ethiopia,” Ethiopian Journal of Health Sciences 31, no. 3 (2021): 515–524.

[10] H. Kloos , A. M. Polderman , G. Desole , and A. Lemma , “ *Haematobium* Schistosomiasis Among Seminomadic and Agricultural Afar in Ethiopia,” Tropical and Geographical Medicine 29, no. 4 (1977): 399–406. 610024

[11] D. E. Nwele , E. N. Afiukwa , C. A. Uhuo , G. A. Ibiam , and N. B. Agumah , “Human Water Contact Activities and Associated Urogenital Schistosomiasis in Nkalagu Community, Ebonyi State, Nigeria,” Nigerian Journal of Parasitology 38, no. 2 (2017): 153.

[12] A. Alemu , A. Atnafu , Z. Addis , et al., “Soil Transmitted Helminths and *Schistosoma Mansoni* Infections Among School Children in Zarima Town, Northwest Ethiopia,” BMC Infectious Diseases 11 (2011): 189. 21740589 10.1186/1471-2334-11-189 PMC3142518

[13] N. Abebe , B. Erko , G. Medhin , and N. Berhe , “Clinico‐Epidemiological Study of *Schistosomiasis Mansoni* in Waja‐Timuga, District of Alamata, Northern Ethiopia,” Parasites & Vectors 7 (2014): 158. 24690404 10.1186/1756-3305-7-158 PMC4022361

[14] J. E. Grimes , D. Croll , W. E. Harrison , J. Utzinger , M. C. Freeman , and M. R. Templeton , “The Roles of Water, Sanitation and Hygiene in Reducing Schistosomiasis: A Review,” Parasites & Vectors 8 (2015): 156. 25884172 10.1186/s13071-015-0766-9 PMC4377019

[15] Z. Mekonnen , S. Meka , A. Zeynudin , and S. Suleman , “Schistosoma Mansoni Infection and Undernutrition Among School Children in Finch'a Sugar Estate, Rural Part of West Ethiopia,” BMC Research Notes 13, no. 1 (2020): 1–6. 25348748 10.1186/1756-0500-7-763 PMC4216851

[16] A. Ali , C. Lo , and T. Ayele , “Schistosoma Haematobium in Western Ethiopia,” Ethiopian Medical Journal 24, no. 2 (1986): 41. 3084236

[17] A. Lemma “Bilharziasis in the Awash Valley. An Epidemiological Study With Special Emphasis on Its Possible Future Economic and Public Health Importance.” (1969): 147–176.

[18] H. B. L. Russell , 1958. Pilot Mobile Health Team, Ethiopia. Assignment Report WHO/EM/PHA/62, Ethiopia 13 of the World Health Organization, Geneva.

[19] Three New Woredas Decided in A Far Region” Archived (https://web.archive.org/web/20081121211708/http://www.afarfriends.org/Dok%20t%20websida/APDA/apda20070204.pdf) November 21, 2008, at theWayback Machine.

[20] Hailu Ejara Kene , Baseline Survey of 55 Weredas of PCDP Phase II, Part I (http://www.pcdp.org.et/Docs/baseline/pcdpII%20%20One.pdf) Archived (https://web.archive.org/web/20110720151420/http://www.pcdp.org.et/Docs/baseline/pcdpII%20-%20One.pdf) July 20, 2011, at the Wayback Machine (Addis Ababa: August 2008), Annex 1 (accessed 23 March) 2009.

[21] N. A. Elsammani , A. Ali Adam , A. A. Mater , and M. O. Elamin , “Prevalence of Schistosomiasis Among School Children in Bahri Locality, Sudan,” International Journal of Research—GRANTHAALAYAH 7 (2019): 299–306.

[22] E. Amuta and R. Houmsou , “Prevalence, Intensity of Infection and Risk Factors of Urinary Schistosomiasis in Pre‐School and School Aged Children in Guma Local Government Area, Nigeria,” Asian Pacific Journal of Tropical Medicine 7, no. 1 (2014): 34–39, Available from: 10.1016/S1995-7645(13)60188-1. 24418080

[23] A. Degarege , Z. Mekonnen , B. Levecke , and M. Legesse , Prevalence of Schistosoma Heamatobium Infection Among School‐Age Children in Afar (2015), 1–11. 10.1371/journal.pone.0133142 PMC4529094 26252615

[24] J. R. Stothard , J. C. Sousa‐Figueiredo , M. Betson , et al., “9789241503174_Eng,” Trends In Parasitology 29, no. 1 (2016): 1–9.

[25] H. Sady , H. M. Al‐Mekhlafi , W. M. Atroosh , et al., “Knowledge, Attitude, and Practices Towards Schistosomiasis Among Rural Population in Yemen,” Parasites & Vectors 8, no. 1 (2015): 436, Available from: 10.1186/s13071-015-1050-8. 26302747 PMC4548916

[26] WHO Expert Committee on the Control of Schistosomiasis . Prevention and Control of Schistosomiasis and Soil‐Transmitted Helminthiasis: Report of a WHO Expert Committee. No. 912. World Health Organization, 2002. 12592987

[27] S. Hussen , D. Assegu , B. T. Tadesse , and T. Shimelis , “Prevalence of Schistosoma mansoni Infection in Ethiopia: A Systematic Review and Meta‐Analysis,” Tropical Diseases, Travel Medicine and Vaccines 7, no. 1 (2021): 4, 10.1186/s40794-020-00127-x. 33522949 PMC7849146

[28] T. Hailegebriel , E. Nibret , and A. Munshea , “Prevalence of *Schistosoma Mansoni* and S*. Haematobium* in Ethiopia: A Systematic Review and Meta‐Analysis,” Tropical Medicine & International Health 26, no. 8 (2021): 886–900.

[29] K. Hajissa , A. E. M. A. Muhajir , H. A. Eshag , et al., “Prevalence of Schistosomiasis and Associated Risk Factors Among School Children in Um‐Asher Area, Khartoum, Sudan,” BMC Research Notes 11, no. 1 (2018): 779. 30382901 10.1186/s13104-018-3871-y PMC6211415

[30] S. Muhumuza , A. Olsen , F. Nuwaha , and A. Katahoire , “Understanding Low Uptake of Mass Treatment for Intestinal Schistosomiasis Among School Children: A Qualitative Study in Jinja District, Uganda,” Journal of Biosocial Science 47, no. 4 (2015): 505–520, 10.1017/S002193201400011X. 24735860

[31] O. G. Otuneme , O. O. Obebe , T. T. Sajobi , W. A. Akinleye , and T. G. Faloye , “Prevalence of Schistosomiasis in a Neglected Community, South Western Nigeria at Two Points in Time, Spaced Three Years Apart,” African Health Sciences 19, no. 1 (2019): 1338–1345, 10.4314/ahs.v19i1.5 31148959 PMC6531954

[32] E. J. Fok , M. Kedir , D. Getachew , and G. Tasew , “Prevalence of *Schistosoma Haematobium* Infection Among School‐Age Children in Afar and Gambella Regions, Ethiopia,” Ethiopian Journal of Health Sciences 31, no. 3 (2021): 515–524.

[33] A. Tsegaye , A. Wossene , W. Awoke , and M. Anberber , “Prevalence of *Schistosoma Haematobium* Infection and Associated Factors Among Preschool Children in Gambella Region, Ethiopia,” Journal of Parasitology Research (2022): 8876543.

[34] W. Awoke , M. Bedimo , and M. Tarekegn , “Prevalence of Schistosomiasis and Associated Factors Among Students Attending at Elementary Schools in Amibera District, Ethiopia,” Open Journal of Preventive Medicine 3, no. 2 (2013): 199–204.

[35] T. E. Atalabi , U. Lawal , and S. J. Ipinlaye , “Prevalence and Intensity of Genito‐Urinary Schistosomiasis and Associated Risk Factors Among Junior High School Students in Two Local Government Areas Around Zobe Dam in Katsina State, Nigeria,” Parasites & Vectors 9, no. 1 (2016): 388. 27388007 10.1186/s13071-016-1672-5 PMC4936285

[36] A. Dabo , A. Z. Diarra , V. Machault , et al., “Urban Schistosomiasis and Associated Determinant Factors Among School Children in Bamako, Mali, West Africa,” Infectious Diseases of Poverty 4, no. 1 (2015): 4. 25973199 10.1186/2049-9957-4-4 PMC4429506

[37] A. Fenwick , J. P. Webster , E. Bosque‐Oliva , et al., “The Schistosomiasis Control Initiative (SCI): Rationale, Development and Implementation From 2002–2008,” Parasitology 136, no. 13 (2009): 1719–1730. 19631008 10.1017/S0031182009990400

[38] K. O. A. Abu Salif , H. S. Abd Allah , M. al‐Tayyib , and K. A. Abd al‐Salam , “Evaluation of Various Techniques Used for the Diagnosis of Schistosomiasis,” Sudan Medical Laboratory Journal 1, no. 2 (2011): 49–59.

[39] B. C. Dazo and J. E. Biles., “Two New Field Techniques for Detection and Counting of Schistosoma Haematobium Eggs in Urine Samples, With an Evaluation of Both Methods,” Bulletin of the World Health Organization 51, no. 4 (1974): 399–408. 4549491 PMC2366293

[40] N. Lodh , J. C. L. Mwansa , M. M. Mutengo , and C. J. Shiff , “Detection of Parasite‐Specific DNA in Urine Sediment Obtained by Filtration Differentiates Between Single and Mixed Infections of Schistosoma Mansoni and S. Haematobium From Endemic Areas in Ghana,” ScienceOpen [Internet] (2014): Mar 13. https://www.scienceopen.com/document/read?-1.ILinkListener-found-article~stats~widget-recommend~link&vid=de22d6fb-2618-4f3f-8e3d-bab4248cb894. 10.1371/journal.pone.0091144 PMC3954594 24632992

[41] P. Mungai , J. C. L. Mulugeta Mwansa , M. M. Mutengo , et al., “Prevalence of Schistosomiasis and Its Impact on the Health of School‐Aged Children in Kenya,” International Journal of Environmental Research and Public Health 15, no. 10 (2018): 2215.30309043

[42] P. Steinmann , J. Keiser , R. Bos , M. Tanner , and J. Utzinger , “Schistosomiasis and Water Resources Development: Systematic Review, Meta‐Analysis, and Estimates of People at Risk,” The Lancet Infectious Diseases 6, no. 7 (2006): 411–425. 16790382 10.1016/S1473-3099(06)70521-7

[43] S. Sow , S. J. De Vlas , D. Engels , and B. Gryseels , “Water‐Related Disease Patterns Before and After the Construction of the Diama Dam in Northern Senegal,” Annals of Tropical Medicine and Parasitology 96, no. 6 (2002): 575–586. 12396320 10.1179/000349802125001636

[44] M. Kabuyaya , M. J. Chimbari , T. Manyangadze , and S. Mukaratirwa , “Schistosomiasis Risk Factors Based on the Infection Status Among School‐Going Children in the Ndumo Area, uMkhanyakude District, South Africa,” Southern African Journal of Infectious Diseases 32, no. 2 (2017): 67–72.

[45] J. E. Grimes , D. Croll , W. E. Harrison , J. Utzinger , M. C. Freeman , and M. R. Templeton , “The Roles of Water, Sanitation and Hygiene in Reducing Schistosomiasis: A Review,” Parasites & Vectors 8, no. 1 (2015): 156. 25884172 10.1186/s13071-015-0766-9 PMC4377019

[46] D. G. Colley , A. L. Bustinduy , W. E. Secor , and C. H. King , “Human Schistosomiasis,” The Lancet 383, no. 9936 (2014): 2253–2264. 10.1016/S0140-6736(13)61949-2 PMC4672382 24698483

[47] N. Berhe , G. Medhin , B. Erko , et al., “Variations in Helminth Faecal Egg Counts in Kato–Katz Thick Smears and Their Implications in Assessing Infection Status With Schistosoma Mansoni,” Acta Tropica 92, no. 3 (2004): 205–212. 15533288 10.1016/j.actatropica.2004.06.011

[48] World Health Organization , 2023, Schistosomiasis Fact Sheet, accessed August 20, 2024. https://www.who.int/news-room/factsheets/detail/schistosomiasis.

